# Physiological and Psychological Responses to a Maximal Swimming Exercise Test in Adolescent Elite Athletes

**DOI:** 10.3390/ijerph18179270

**Published:** 2021-09-02

**Authors:** Gábor Almási, Edit Bosnyák, Ákos Móra, Annamária Zsákai, Piroska V. Fehér, Dorina Annár, Nikoletta Nagy, Zsófia Sziráki, Han C. G. Kemper, Márta Szmodis

**Affiliations:** 1 Department of Health Science and Sports Medicine, University of Physical Education, 1123 Budapest, Hungary; bosnyak.edit@tf.hu (E.B.); szirakizsofi@gmail.com (Z.S.); szmodis.marta@tf.hu (M.S.); 2 Faculty of Health Sciences, University of Pécs, 7621 Pécs, Hungary; akosmora86@gmail.com; 3 Department of Biological Anthropology, Eötvös Loránd University, 1117 Budapest, Hungary; annamaria.zsakai@ttk.elte.hu (A.Z.); feh.pir@gmail.com (P.V.F.); annar.dorina@gmail.com (D.A.); 4 Department of Swimming and Water Sports, University of Physical Education, 1123 Budapest, Hungary; nagy.nikoletta@tf.hu; 5 EMGO Institute for Health and Care Research, Amsterdam University Medical Center, 1081 Amsterdam, The Netherlands; hancgkemper@upcmail.nl

**Keywords:** adolescent elite, physiology, psychology, overtraining, swimming

## Abstract

Background: Continuously rising performances in elite adolescent athletes requires increasing training loads. This training overload without professional monitoring, could lead to overtraining in these adolescents. Methods: 31 elite adolescent athletes (boys: *n* = 19, 16 yrs; girls: *n* = 12, 15 yrs) participated in a field-test which contained a unified warm-up and a 200 m maximal freestyle swimming test. Saliva samples for testosterone (T) in boys, estradiol (E) in girls and cortisol (C) in both genders were collected pre-, post- and 30 min post-exercise. Lactate levels were obtained pre- and post-exercise. Brunel Mood Scale, Perceived Stress Scale and psychosomatic symptoms questionnaires were filled out post-exercise. Results: Lactate levels differed between genders (boys: pre: 1.01 ± 0.26; post: 8.19 ± 3.24; girls: pre: 0.74 ± 0.23; post: 5.83 ± 2.48 mmol/L). C levels increased significantly in boys: pre- vs. post- (*p* = 0.009), pre- vs. 30 min post-exercise (*p* = 0.003). The T level (*p* = 0.0164) and T/C ratio (*p* = 0.0004) decreased after field test which draws attention to the possibility of overtraining. Maximal and resting heart rates did not differ between genders; however, heart rate recovery did (boys: 29.22 ± 7.4; girls: 40.58 ± 14.50 beats/min; *p* = 0.008). Conclusions: Our models can be used to explain the hormonal ratio changes (37.5–89.8%). Based on the results this method can induce hormonal response in elite adolescent athletes and can be used to notice irregularities with repeated measurements.

## 1. Introduction

The high physiological and psychological demand of physical exercise and training with maximal intensity creates short- and long-term responses and adaptations of the human body. The well-known morphological and cellular changes are observed of inadequate adaptation to exercise in adults [1]. However, knowledge of adolescent elite athletes is still limited in this field of sport sciences.

Hormonal changes induced by exercise and training have an important role in adaptation, especially among adolescent athletes due to the higher level of hormone production through maturation. Hormones affect body composition through energy balance, cellular structure changes, mineral absorption and secretion, responses to stimulus, function of the immune system and several other processes through inhibition or stimulation [2].

Research indicates that cortisol (C) and androgens are the most sensitive hormones to stress. Several studies investigated the effect of exercise and psychological stress to C level in adults. In most cases, the C level after training or competition was higher than the basal level [3,4,5,6,7]. The change is described by the stress-induced secretion of C as physical exercise is a stressor for the human body.

Dehydroepiandrosterone (DHEA) and dehydroepiandrosterone sulphate (DHEA-S) are two of the major steroid hormones of the adrenal gland, and the primary precursor of estradiol (E). These also appeared in numerous studies conducted with the participation of adult and adolescent female athletes. There are studies where DHEA and DHEA-S levels did not differ after exercise from the resting values in adults [5,8]. In one of the few studies with adolescent participants, DHEA-S basal level increased after physical exercise [9]. Estradiol production also reacts to physical exercise. Researchers recorded an exercise-related increase of E level in females; however, the rise was only significant in the mid-luteal phase [10]. Other studies showed that heavy endurance training can disrupt the menstrual cycle and E production in adult females [11,12].

As the current literature declares, testosterone (T) measurement in females does not show significant changes to physical exercise due to the low T concentration, which indicates the usage of a different marker, such as E. In contrast, T level changes in males can be a reliable marker of physiological changes triggered by physical exercise. Studies show that high demanding prolonged exercise (such as long-distance running) decreases the free T level; however, various other types of exercise show no changes in free T level in adult males [13,14]. There are a few studies of acute exercise-induced steroid hormonal changes during pubertal age in boys and girls; however, the results are conflicting [15,16].

The changes of lactate (LAC) levels are important physiological factors to monitor the athletes’ training load, recovery and overall fitness. The literature suggests that adult swimmers reach their maximal LAC level (average of 15–16 mmol/L) during the 100 and 200 m events [17]. Adolescent and adult athletes reach their highest LAC level with maximal intensity exercise [18]; therefore, this field test (FT), which is explained in the methods section of this study, seems to be the optimal choice to analyse the effect of maximal exercise on adolescent athletes. In addition, youth elite athletes show high variability of LAC changes depending on gender and age [15].

Psychological responses and adaptation are also important aspects of an athlete’s performance. Several studies have pointed out that mental capabilities play a key role in athletes’ efficiency. The necessity of monitoring the psychological state and its changes in athletes was discovered decades ago as one of the main influencing factors of performance. Researchers described a tendentious change of mood state in adult 400 m swimmers through a season [19]. As the workload increased from the start to the mid-season, negative aspects such as depression, fatigue and anger scores increased, whereas vitality decreased. Overall Profile of Mood State (POMS) scores also increased in the mid-season, and as the workload decreased, the scores reverted close to the original results. Staleness is also a recurring behaviour in adolescent athletes, as 30–48% of them reported being stale at least once in a season [20]. The effect of these factors and their correlation with physiological changes are also well studied [19,20] and sometimes incorporated into adult athletes’ training, however they are underrepresented among adolescent athletes; however, there are tendentious differences in their mood states [21].

Data suggest there are general changes in given markers induced by physical exercise. Although in some cases these changes diverge from the common trend. Sometimes these changes might be caused by overtraining (OT) or overtraining syndrome (OTS). As studies suggests, 10 to 20% of athletes’ experience OTS or staleness. Some studies characterise eight common OTS symptoms including poor performance, prolonged recovery, disturbed mood states, elevated resting pulse, etc. [1]. The effect of too high intensity training through a prolonged period of time can cause this maladaptation, resulting in a lower level of performance, disturbed physiological and psychological states and adaptations [20,22,23,24]. As these states result in disrupted responses to exercise, regular complex measurements of these physiological and psychological markers might be the solution to diagnose OT and OTS in an early stage, resulting in shorter recovery time.

Adolescent athletes have to face and cope with a growing level of physical and mental expectations which require higher amounts of training. If the training load is not chosen carefully, then it can lead to maladaptation and poor or deteriorating physiological and psychological condition or illness, which is one of the main reasons for early burnout and drop out. Currently, to the best of our knowledge, there is no method for youth athletes, which is easily and efficiently usable during training session that could alert the athletes and coaches of inadequate training and early symptoms of OT.

Although cortisol and androgens are the most sensitive hormones for detecting exercise-induced stress and it is well-researched among adult athletes, the literature focusing on adolescent athletes is limited. Inspired by the limited literature of youth layer our study focuses on the uniform exercise-induced physiological and psychological changes of elite adolescent athletes.

We believe that using hormonal measurements, psychological questionnaires and physiological monitoring, it is possible to compile a simple and efficient method that can be used by coaches to diagnose the first symptoms of OT before its development. This could prove extremely useful in determining training loads individually for the athletes to achieve a long, successful and healthy sport career.

## 2. Materials and Methods

### 2.1. Participants

This study was conducted with the participation of 31 adolescent water sport (swimming and water polo) athletes (boys: *n* = 19, mean age ± SD: 16.34 ± 1.12 years; girls: *n* = 12, 15.17 ± 0.81 years) from Hungary. The different age criteria between the genders were deliberately chosen due to the difference in the two genders’ biological maturing as the girls reach the same Tanner stage in younger age than boys. The following criteria were used to select the elite athletes: at least 10 h of weakly training, regular participation in national and international competitions, at least 5 years of sport-related exercise and no excessive fatigue or influencing medical condition at the time of testing. Written parental consent and the children’s assent were collected prior to the investigation. All participants received written and verbal information about the aim of the survey and the procedures used. 

### 2.2. Swimming Test

After the thorough review of the existing literature and consultations with several water sport coaches a swimming test protocol was created. The FT (field test) took place in the winter of 2018/2019 in the afternoon from 15:00 to 17:00 and contained the following phases: prior to the swimming test, the athletes executed a 25 min general, uniform warm up with varying intensity planned and lead by the same coach. After the warming-up session, participants rested for 10 min before the maximal effort. The rest contained active and passive stretching exercises. To ensure hormonal response to the exercise, the 200 m freestyle event was chosen [17], and the participant had to swim at maximal effort which was followed by a resting phase where the LAC measurements took place. 

### 2.3. Hormonal Measurements

To measure the changes in salivary T, E and C we used IBL C Saliva ELISA Kit (RE52611, Tecan Group Ltd., Männedorf, Switzerland). The saliva samples were collected before the warmup for pre-exercise level (T_pre_; E_pre_; C_pre_), after the 200 m maximal swimming (C_post_) and 30 min later (T_post30;_ E_post30_; C_post30_) to measure the hormonal responses to the FT. The samples were collected, stored and analysed according to the manufacturer’s guidelines. A base level of cortisol was achieved by avoiding physical stress (physical activity, significant changes of environmental parameters as temperature, humidity, etc.) and emotional stress (fear, nervousness, panic, etc.) one hour before and during the saliva collection. By following the kit protocol and recommendations subjects were asked to avoid eating, drinking, chewing gum or brushing their teeth for 30 min before sampling. Saliva samples were stored at −20 °C (no longer than 2 weeks before the assays), warmed up to room temperature and centrifuged to separate the mucins. All standards, samples and controls were run in duplicate, the absorbance of each well was determined with an Epoch 267860 microtiter plate reader at 450 nm.

Females’ saliva samples were analysed for E and C level, while males’ samples were tested for T and C level.

### 2.4. Lactate Measurements

LAC measurements of the athletes were performed pre- and post-FT. Samples were collected from their earlobe. Basal (LAC_pre_) levels were achieved in the afternoon, before the FT. The participants did not take part in any training course on the day of the testing, and they were asked to refrain from any other physical activity during the day. Blood LAC measurements after the FT (LAC_post_) were conducted from 60 s after the completion of the 200 m swimming to capture the highest concentration of LAC of each athlete. We used Lactate Plus Sport Meter (Nova Biomedical, Waltham, MA, USA) to measure LAC level.

### 2.5. Heart Rate Measurements

The subjects’ heart rates (HR) were recorded during the swimming test and the following 60 s of recovery by Polar V800 HR monitor (Polar Electro BV, Kempele, Finland) with an attached Polar H10 transmitter. Additional straps over the athletes’ shoulder were used to ensure that the sensor stays in place throughout the whole FT. After obtaining the data from the monitors the average resting HR (HRrest), the maximal HR during the FT (HRmax), the difference between the HR at the end of the maximal swimming and 60 s after the end of maximal swimming (HRR60) and the ratio of and the athletes’ calculated possible maximal HR and HRmax (HR% = $\frac{220-their\;current\;age}{HRmax}$) were determined.

### 2.6. Psychological Tests

The subjects completed three questionnaires to assert their current and overall mental states. The BRUNEL mood scale (BRUMS) [25,26] was used to determine the current mood state of the participants. The Hungarian version of the mood scale was translated and validated (Cronbach-alpha: 0.70–0.89) [27]. It contains 32 questions with 5 points on the Likert scale as possible answers. The resulting 6 mood items’ scores are calculated from the answers: Anger (Bang), Confusion (Bconf), Depression (Bdep), Fatigue (Bftg), Tension (Btens) and Vigour (Bvig). The Perceived Stress Scale’s (PSS) [28] was used to assess last month’s level of perceived stress of the participants contains 14 items, the Hungarian version was created validated (Cronbach-alpha: 0.88) [29]. The result of the PSS scale (PSSsum) was calculated by the guidelines of the questionnaire. The frequency of the psychosomatic symptoms (PSsympt) (Cronbach-alpha: 0.81) over the past six months was assessed by another questionnaire with 12 items [30]. These questionnaires also have 5 possible answers with a Likert scale. The subjects were asked to fill these out after the 200 m maximal swimming test. 

### 2.7. Statistical Analysis

For the statistical analysis, the Statistica for Windows 13 software (TIBCO Software Inc., Palo Alto, CA, USA) was used. The normality of the data was determined, the analysed data followed a normal distribution Kolmogorov–Smirnov test. The following methods were used:

- descriptive statistics (mean ± SD),
- Student *t*-test for independent samples to analyse gender differences
- Student *t*-test for dependent samples to analyse pre and post exercise differences
- Pearson’s linear correlation analysis to show associations of different variables
- Linear multiple regression analysis to predict hormonal changes as OT indicators

One of the regression models was based on physiological considerations while the other was created by the forward stepwise method. Results were accepted as significant with fewer than 5% of random error [31]. The recommendations of Declaration of Helsinki were conformed, and the ethical approval was obtained from the ethical committee of the University of Physical Education, Hungary (ID:TE-KEB/No10/2019). All data were stored securely and only the relevant researchers had access to it.

## 3. Results

The comparison between genders revealed several, significant differences. According to the selection criteria, the average calendar age of the boys was higher than that of the girls (16.3 ± 1.1 years vs. 15.2 ± 0.8 years; *p* = 0.0058). However, we could not find a difference between the two genders in Tanner stage (girls: 3.6 ± 0.5; boys: 4.0 ± 0.8), sport related experience (girls: 7.0 ± 1.2 yrs; boys: 8.6 ± 2.4 yrs) and training hours per week (girls: 16.9 ± 4.7 h; boys: 14.6 ± 5.8 h).

Several physiological changes and differences among the results of boys and girls were found. Boys’ T level was elevated post-exercise compared to pre-exercise levels; however, girls’ E level post-FT did not differ significantly from the pre-FT level (Table 1). Boys’ T/C ratio also decreased from pre-FT level, while girl’s E/C ratio did not change (Table 1). The LAC_post_ levels were significantly higher than LAC_pre_ in both genders, moreover LAC_pre_ and LAC_post_ levels were higher among boys than girls (Table 1).

Although the level of C before the test (C_pre_) showed higher mean values in girls than boys (0.33 ± 0.09 vs. 0.17 ± 0.13 µg/dL), this difference was not significant (*p* = 0.057).

**Figure 1:**
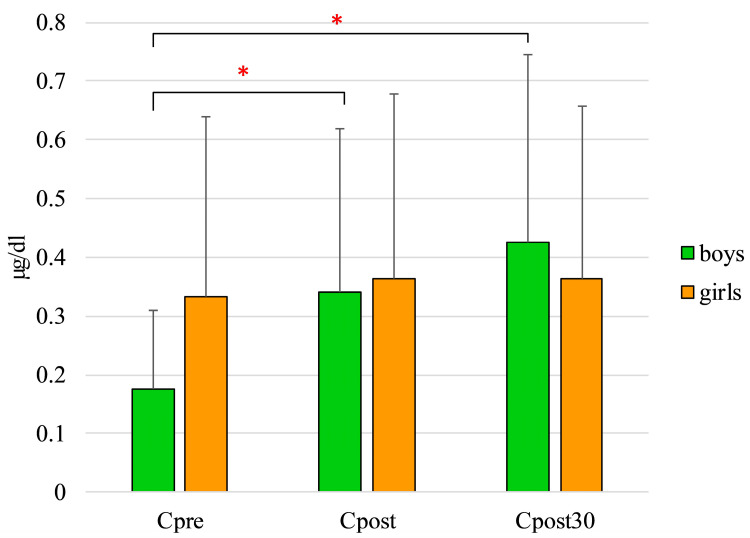
C level changes of boys and girls (* *p* < 0.05). Cpre: cortisol levels before the field test; Cpost: cortisol levels after the field test; Cpost30: cortisol levels 30 min after the field test.

The C level in boys after the maximal exercise swim test (C_post_; 0.340 ± 0.0.278 µg/dL; *p* = 0.009) and 30 min later (C_post30_: 0.426 ± 0.319 µg/dL; *p* = 0.003) were significantly higher than C_pre_ level. The experienced small increase in girls was not significant (C_pre_: 0.333 ± 0.0.30 vs. C_post_: 0.364 ± 0.315 µg/dL) with remarkable individual differences (Figure 1).

**Figure 2:**
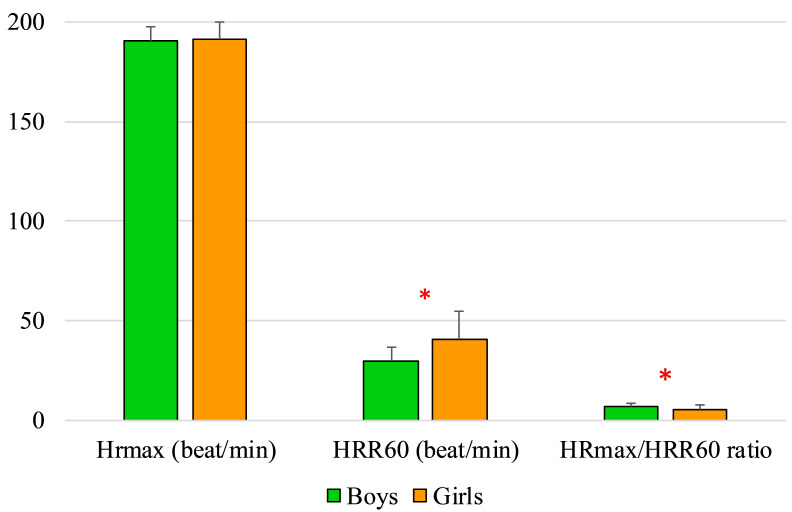
Boys’ and girls’ heart rate changes (* *p* < 0.05). HRmax: maximal heart rate during the maximal exercise; HRR60: difference between the heart rate at the end of the 200 m freestyle swimming and 60 s later; HRmax/HRR60: ratio of maximal heart rate during the 200 m maximal freestyle swimming and the difference between the heart rate at the end of the 200 m freestyle swimming and 60 s later.

The maximal heart rate during the FT did not differ between boys and girls (190.278 ± 7.537 vs. 190.917 ± 9.040 beat/min); however, the HRR60 was higher in girls (29.22 ± 7.37 vs. 40.583 ± 14.501 beat/min; *p* = 0.0084), whereas the HRmax/HRR60 ratio was higher in boys (6.861 ± 1.542 vs. 5.388 ± 2.314; *p* = 0.045) (Figure 2).

There were no significant differences between genders in the PSSsum (girls: 23.17 ± 6.58, boys: 20.89 ± 7.77) and PSsympt (girls: 15.33 ± 7.61, boys: 13.26 ± 6.05). The result points of the BRUNEL questionnaires were the following: Bang girls: 56.42 ± 10.83, boys: 55.42 ± 10.75; Bconf girls: 61.00 ± 12.93, boys: 51.63 ± 7.80 *; Bdep girls: 51.25 ± 7.09, boys 51.95 ± 8.68; Bftg girls: 52.58 ± 8.97, boys: 57.42 ± 10.85; Btens girls: 46.25 ± 6.58, boys 46.21 ± 8.45; and Bvig girls: 53.67 ± 7.40, boys: 52.05 ± 7.38. There was only one significant difference among genders in the BRUNEL scale’ moods: it was in the Confusion (Bconf) subscale, wherein girls’ score was higher than boys’ (*p* = 0.017).

**Figure 3:**
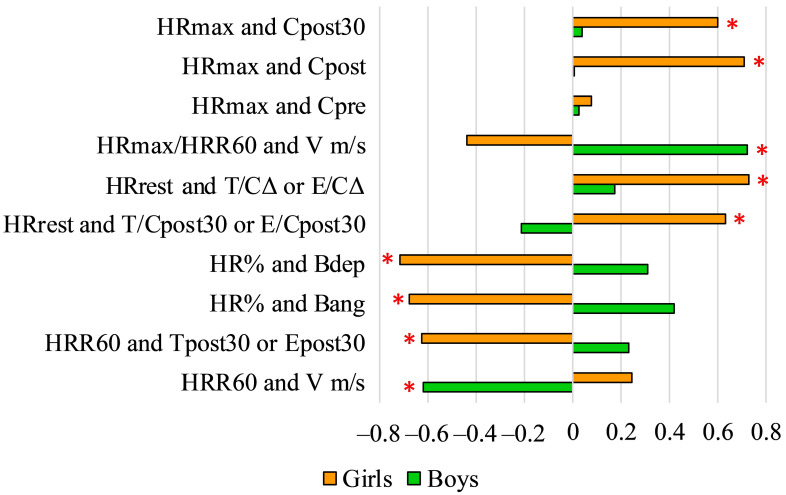
Pearson’s correlation coefficient (r) values of HR variables and other psychological and physiological variables (* *p* < 0.05). HRmax: maximal heart rate during the maximal exercise; Cpost30: cortisol levels 30 after the field test; Cpost: cortisol levels after the field test; Cpre: cortisol levels before the field test; HRmax/HRR60: ratio of maximal heart rate during the 200 m maximal freestyle swimming and the difference between the heart rate at the end of the 200 m freestyle swimming and 60 s later; V m/s: average speed of the swimmers’ during the 200 m maximal freestyle swimming; HRrest: resting heart rate; T/CΔ: difference of testosterone-cortisol ratio before the field test and 30 min after the field test; E/CΔ: difference of estradiol-cortisol ratio before the field test and 30 min after field test; T/Cpost30: testosterone/cortisol ratio 30 min after the field test; E: estradiol; E/Cpost30: estradiol/cortisol ratio 30 min after the field test; HR%: the ratio of and the athletes’ calculated possible maximal heart rate and maximal heart rate during the maximal exercise; Bang: score of anger on the BRUNEL scale; HRR60: difference between the heart rate at the end of the 200 m freestyle swimming and 60 s later; Tpost30: testosterone levels 30 min after the field test; Epost30: estradiol levels 30 min after the field test.

We found several significant correlations between physiological, performance and psychological parameters in both boys and girls, and remarkable gender differences. Girls’ maximum HR during swimming (HRmax) correlated significantly with both C_post_ and C_post30_ levels, whilst there were no correlations in this respect in boys. Heart rate variables (HRmax/HRR60 ratio, HR%, HRrest and HRR60) also correlated with different variables in boys and girls (Figure 3).

**Figure 4:**
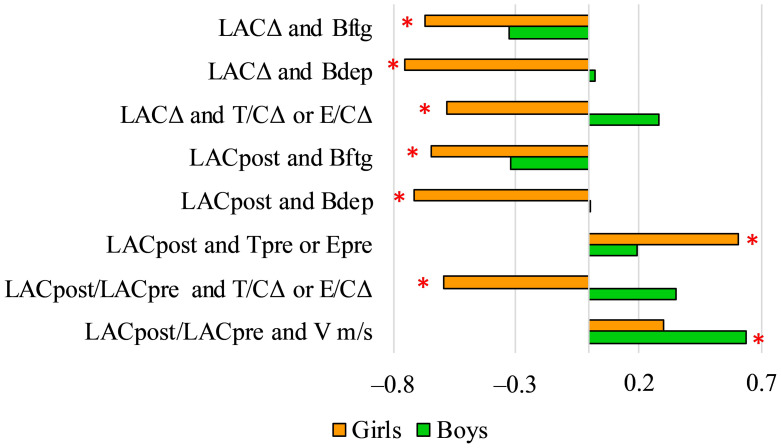
Pearson’s correlation coefficient (r) values of LAC variables and other psychological and physiological variables (* *p* < 0.05). LACΔ: difference of lactate levels before and after the field test; Bdep: score of depression on the BRUNEL scale; T/CΔ: difference of testosterone-cortisol ratio before the field test and 30 min after the field test; E/CΔ: difference of estradiol-cortisol ratio before the field test and 30 min after field test; Bftg: score of fatigue on the BRUNEL scale; LACpost: lactate levels after the field test; Tpre: testosterone levels before the field test; Epre: estradiol levels before the field test; LACpost/LACpre: ratio of lactate levels before and after the field test; V m/s: average speed of the swimmers’ during the 200 m maximal freestyle swimming.

The LAC_post_ levels, the difference between LAC_pre_ and LAC_post_ levels (LAC_Δ_) and LAC_post_/LAC_pre_ correlated with different variables in boys and girls (Figure 4).

**Figure 5:**
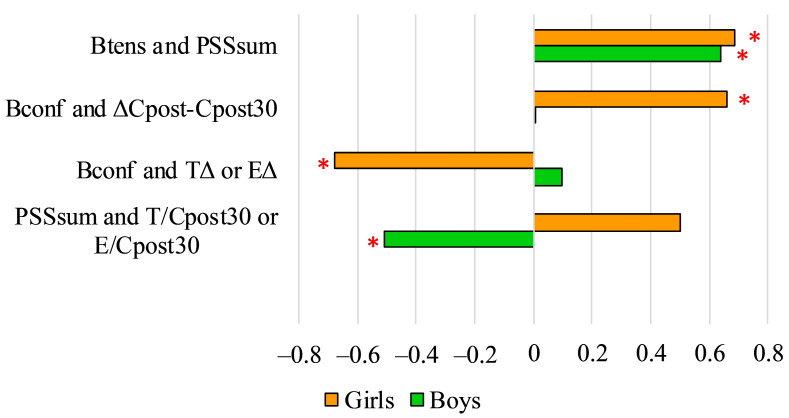
Pearson’s correlation coefficient (r) values of PSS and BRUNEL questionnaire items and other psychological and physiological variables (* *p* < 0.05). Btens: score of tension on the BRUNEL scale; Bconf: score of confusion on the BRUNEL scale; PSSsum: sum of Perceived Stress Scale scores; ΔCpost-Cpost30: C difference of cortisol levels after the field test and 30 min after the field test; TΔ: difference of testosterone levels before the field test and 30 after the field test; EΔ: difference of estradiol levels before the field test and 30 after the field test; T/Cpost30: testosterone/cortisol ratio 30 min after the field test; E: estradiol; E/Cpost30: estradiol/cortisol ratio 30 min after the field test.

The results of PSS and BRUNEL questionnaires also showed significant correlations with other variables (Figure 5).

The multiple regression models for E/C_post30_ revealed significant relations of steroid hormones with different lactate levels, HR data and psychological characteristics. The girls’ first model by certain physiological consideration consists of LAC_Δ_ and HRrest. The second forward regression model consists of HRmax, HRrest, LAC_post_/LAC_pre_ ratio, Bvig and PSsympt. Boys’ first model for T/C_post30_ consists of HRR60 and LAC_post_/LAC_pre_ ratio. The second model consists of HRR60 and LAC_post_/LAC_pre_ ratio, HRrest, PSSsum, Btens and Bftg (Table 2).

## 4. Discussion

The significant difference in calendar age between the genders met our requirements, and the lack of difference in Tanner stages shows that the selecting criteria were determined sufficiently, which was an important part of this investigation due to the hormonal aspects.

LAC level demonstrated the expected changes to a maximal exercise as it rose in both genders; however, the significant difference between boys and girls was unexpected. As both genders reached similar HRmax values in the field test, we assumed they reached the same level of intensity during the exercise, yet their LAC_post_ levels differed significantly. As research suggests, girls’ LAC level can be influenced by the menstrual phase [32]; however, we did not find correlations between menstrual phases (50% of the girls were in luteal and 50% were in follicular phase) and LAC level. The difference between girls and boys might be caused by the well-known gender difference in body muscle mass and percentage as boys have higher absolute and relative muscles values. Another explanation might be the difference in training methods, which could have led to a higher level of fatigue among older boys, resulting in higher LAC_post_ level [18].

The hormonal changes in boys were conflicting. The C level changed as we expected and as it is suggested by research [3,4,5,6,7]. The result showed a significant and progressive rise which is comprehensible due to the hormonal responses to a stressor, such as a maximal exercise. However, the T level changed contrary to our expectations based on earlier studies [2,33,34], reducing from T_pre_. In the light of these results the decrease in T/C values is understandable; however, as mentioned in research focusing on OT in adult athletes [19,20,23,24], this is not the expected or healthy response to exercise [35]. In contrast to adults, children and adolescent elite athletes’ hormonal responses to exercise is rarely studied in relations to OT and OTS. These are key factors in early burnout which can be associated with C levels [36].

Girls’ hormonal responses after the maximal swimming test showed no significant differences from resting level in contrast to the boys’ results. Nevertheless, the high level of C_pre_ was already unexpected, which may limit further rise influenced by the field test, leaving the question behind of what could have caused the high C_pre_ level. As cortisol’s connection with stress is well-known, the high level of stress might be the cause of high levels of C_pre_ among girls [35]. No significant changes were recorded in E level; however, both C and E level showed remarkable individual differences, which might be the explanation for the lack of significant changes. Another explanation might be the different menstrual stages the participating girls were in which could also affect the hormonal level [37].

As both genders had similar HRmax and HRrest values, the HRR60 and HRmax/HRR60 results showed significant differences between them. Monitoring HR values can sufficiently inform the athletes and coaches about the individual performance, the effectiveness of the trainings and the balance of work/rest ratio. As the literature suggests, there is no concurrent proof of gender differences in HRR60 and HRmax [38,39]. In light of HRmax values the difference is unlikely caused by possible difference in performance during the field test. The difference might be caused by the variance in the athletes training programme which could lead to a different overall fitness state. The high HRR60 results are associated with better adaptation to physical exercise while lower results could indicate chronic fatigue.

Only one of the various mood states showed significant difference between genders as boys had lower scores in the BRUNEL scale confusion subscale. As generally assumed that adolescent girls had higher level of stress compared to boys [40] which could lead to higher level of confusion among them. BRUNEL mood states correlated negatively with physiological factors connected to the individual level of girls’ performance which could mean that girls’ performance might be more influenced by emotional state than boys’.

The occurrence of psychosomatic symptoms more than once a week were tendentiously lower among this study’s participants compared to the representative (R) average of WHO’s 2017/18 report [41] except for irritable, nervous and sleep difficulties in boys: headache: R girls: 35% vs. 27%, R boys: 13% vs. 7%; stomach ache: R girls 22% vs. 0% R boys 9% vs. 0%; backache: R girls: 23% vs. 17%, R boys: 15% vs. 11%; feeling low: R girls: 39% vs. 25%, R boys: 23% vs. 16%; irritable: R girls: 35% vs. 8%, R boys: 20% vs. 21%; nervous: R girls: 45% vs. 42%, R boys 31% vs. 42%; sleep difficulties: R girls: 33% vs. 8%, R boys 16% vs. 26%; dizzy R girls: 18% vs. 8%, boys: 6% vs. 5%.

The results of correlation analysis between cardiac, metabolic, hormonal, psychological and performance variables resulted in different correlation patterns for boys and girls. Girls showed more internal relationships than boys. Girls’ patterns contained more psychological variables than boys which might be related to the different level of stress described by Meiser et al. [42]. The two multiple regression models for each gender were created after the analysis of correlations. The dependent variable was chosen due to the suggestions of the literature in OT as T/C ratio is commonly studied in relations with OT but only in adults. The first regression model (two independent variables) explained 66.1% of E/C_post30_ in girls and 37.5% of T/C_post30_ in boys after FT, while the forward stepwise models explained 89.8% of hormonal ratio in girls and 66.1% in boys.

According to our current knowledge, no other study researched the connections of steroid hormones and physiological and psychological variables; therefore, no comparisons can be made to these results. The high values of R^2^ show that this simple method (HR, LAC and psychological monitoring) can provide useful information on the alerting changes in steroid hormone reaction induced by exercise among elite adolescent athletes.

Our study was the first complex investigation of hormonal, cardiac and psychological changes to maximal intensity stress in adolescent elite athletes (swimmers and water polo players) which focused on variables of physiology (heart rate changes, hormonal response and lactate level changes) and psychology (perceived stress, psychosomatic symptoms and mood states). This could be a milestone to understand the importance of regular monitoring of elite adolescent athletes’ response to exercise. A multi-disciplinary approach could prove that it is useful to repeatedly measure the changes because of the multiple aspects and prolonged development of overtraining, hence the reason for the numerus variables used in this study.

## 5. Conclusions

The developed 200 m maximal freestyle swimming test showed an adequate stimulus to trigger hormonal changes in the adolescent elite swimmers. Therefore, it could be a base to create a system to notice symptoms of inadequate adaptation by repeated measurements and accordingly alter and personalise the adolescent athletes’ dynamic training course. Further investigations could prove useful information about OT with more participants in different sports and repeated measurements making the method even more precise. The numerous connections between physiological and psychological variables proves, that a multidisciplinary approach could be the next step to understanding the principles of the athletes’ performance.

The importance of monitoring and diagnosing OT is without question. The athletes and coaches must be aware of the importance of adequate exercise/rest ratio in training making it essential to be able to notice any signs of inadequate adaptation especially among adolescents. This study has some limitations such as the medium-sized sample, difference in participant numbers in genders and the high intrapersonal variability such as the different menstrual cycles among girls; however, the OT is a serious problem in adolescent athletes. It plays a major part in shortened sport careers, early burnouts and sport-related injuries and illnesses. The provided multiple regression models in this research can maybe become the base of a new alerting method due to its’ simplicity and low requirement of tools. A repeatedly used detailed physiological examination combined with psychological methods can provide indispensable data of the elite adolescent athletes’ workload capacity to avoid over- or under-training.

## Figures and Tables

**Table 1:** Lactate and hormonal changes before and after the field test (FT).

	Boys				
	Pre-FT	SD	Post-FT	SD	*p*
T * (µg/dL)	0.006	0.003	0.005	0.002	*0.0164*
T/C ***	0.047	0.032	0.020	0.015	*0.0004*
LAC *** (mmol/L)	1.011	0.259	8.189	3.237	*>0.0001*
	**Girls**				
	Pre-FT	SD	Post-FT	SD	*p*
E (µg/dL)	0.0003	0.0001	0.0004	0.0001	0.1062
E/C	0.0016	0.0010	0.0021	0.0025	0.5135
LAC *** (mmol/L)	0.742	0.228	5.833	2.476	*>0.0001*
	**Girls**	SD	**Boys**	SD	*p*
LAC_pre_ *	0.742	0.228	1.021	0.255	*0.0043*
LAC_post_ *	5.833	2.476	8.189	3.237	*0.0417*

LAC: lactate level; T: testosterone; T/C: testosterone/cortisol ratio; E: estradiol; E/C: estradiol/cortisol ratio; LACpre: pre-FT lactate level; LAC_post_: post-FT lactate level; ***: significant at the level of *p* < 0.001; *: significant at the level of *p* < 0.05 (*italic*).

**Table 2:** Multivariate analysis of girls’ and boys’ E/C or T/C ratios after the exercise.

Girls E/C_post30_							
M 1	LAC_Δ_	HRrest.					R^2^
B *	−0.51 *	0.61 *					0.661 *
M 2	HRmax	LAC_post_/LAC_pre_	HRrest	Bvig	PSsympt		R^2^
B *	−0.44 *	−0.32	0.64 *	0.28	0.21		0.898 *
**Boys T/C_post30_**							
M 1	HRR60	LAC_pre_/LAC_post_					R^2^
B *	0.73 *	0.43					0.375 *
M 2	HRR60	LAC_pre_/LAC_post_	HRrest	PSSsum	Btens	Bftg	R^2^
B *	0.96 *	0.51	−0.45	−0.49	0.62	−0.41	0.661 *

M: model; T/C_post30_: testosterone/cortisol ratio 30 min after the field test; E: estradiol; E/C_post30_: estradiol/cortisol ratio 30 min after the field test; M: multiple regression model; HRmax: maximal heart rate during the maximal exercise; HRR60: difference between the heart rate at the end of the 200 m freestyle swimming and 60 s later; HRrest: resting heart rate; LAC_pre_/LAC_post_: ratio of lactate levels before and after the field test; LAC_Δ_: difference of lactate levels before and after the field test; Bvig: score of vigour on the BRUNEL scale; Bftg: score of fatigue on the BRUNEL scale; Btens: score of tension on the BRUNEL; PSSsum: sum of Perceived Stress Scale scores; PSsympt: frequency of the psychosomatic symptoms; *: significant result.
